# Supplementary material for: Epigenetic profiling for the molecular classification of metastatic brain tumors
Source: Nat Commun. 2018 Nov 6;9:4627. doi: 10.1038/s41467-018-06715-y (PMC6219520; doi:10.1038/s41467-018-06715-y)
Supplement: Supplementary file 1 — Supplementary Information [file 41467_2018_6715_MOESM1_ESM.pdf]

**Supplementary Information**

Orozco *et al.*

# Epigenetic Profiling for the Molecular Classification of Metastatic Brain Tumors

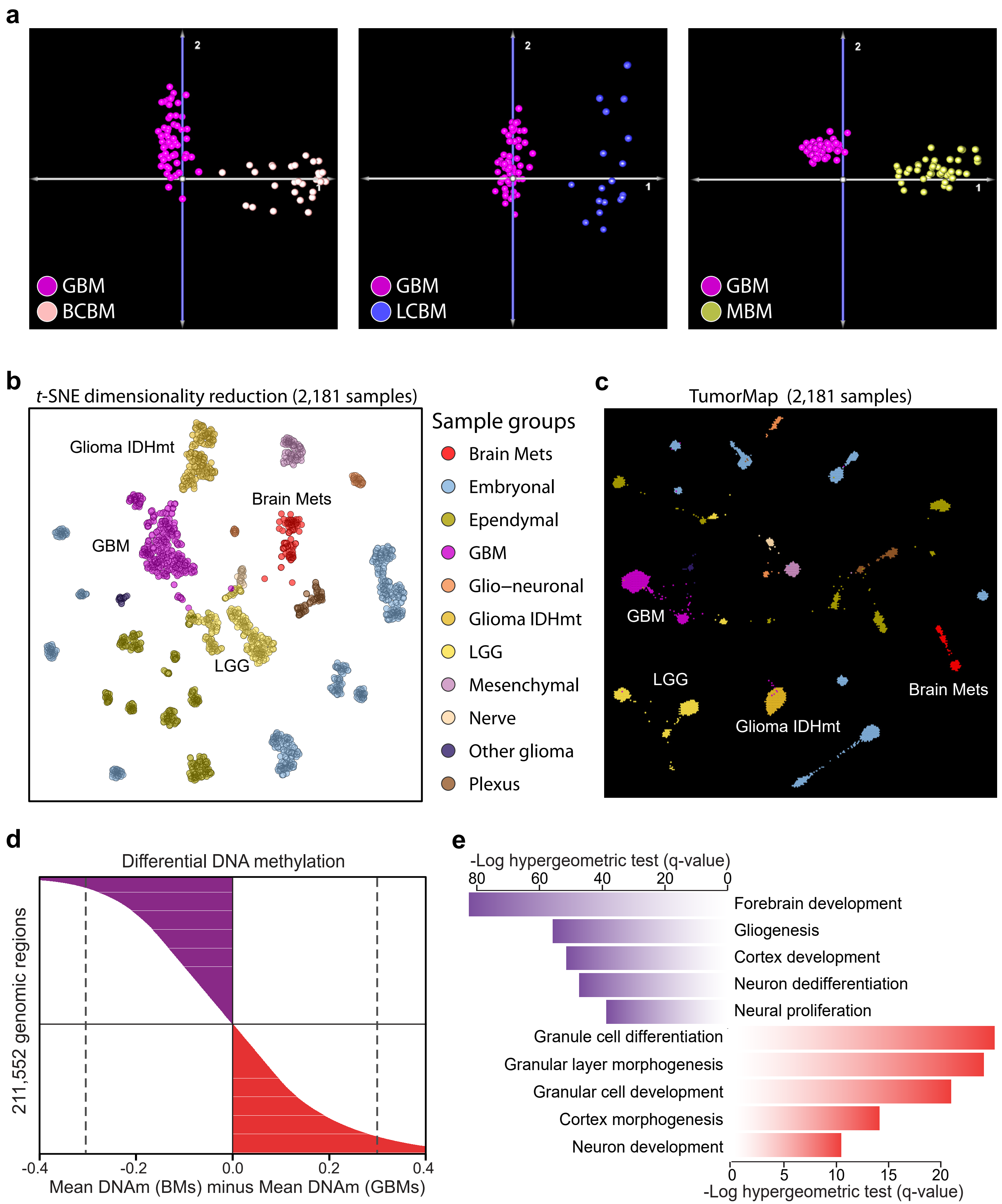


**Supplementary Figure 1**

**Supplementary Figure 1:** a- Principal component analyses (PCA) comparing GBM specimens (n=60) with each of the brain metastasis types (BCBM, LCBM, and MBM; n=96). b and c- Multidimensionality reduction of the entire brain metastasis cohort (n=96), along with a reference cohort of primary central nervous system (CNS) tumors (GSE90496, n=2,085), was performed using the *t*-Distributed Stochastic Neighbor Embedding (t-SNE) technique (b) and the UCSC TumorMap visualization tool (c). Color patterns for all the specimens were matched between the two approaches. d- Differential DNA methylation between GBM and BM specimens. Mean β-values of 211,552 genomic regions in brain neoplasm specimens were compared between groups. Grey dashed lines demarcate genomic regions with at least 30% differential DNA methylation between the groups. e- Biological processes significantly enriched for genes with proximal (up to 5 kb) and distal (up to 500 kb) hypomethylated regions in GBM specimens (purple bars) and hypomethylated regions in BM specimens (red bars), performed using the Genomic Regions Enrichment Annotations Tool (GREAT).

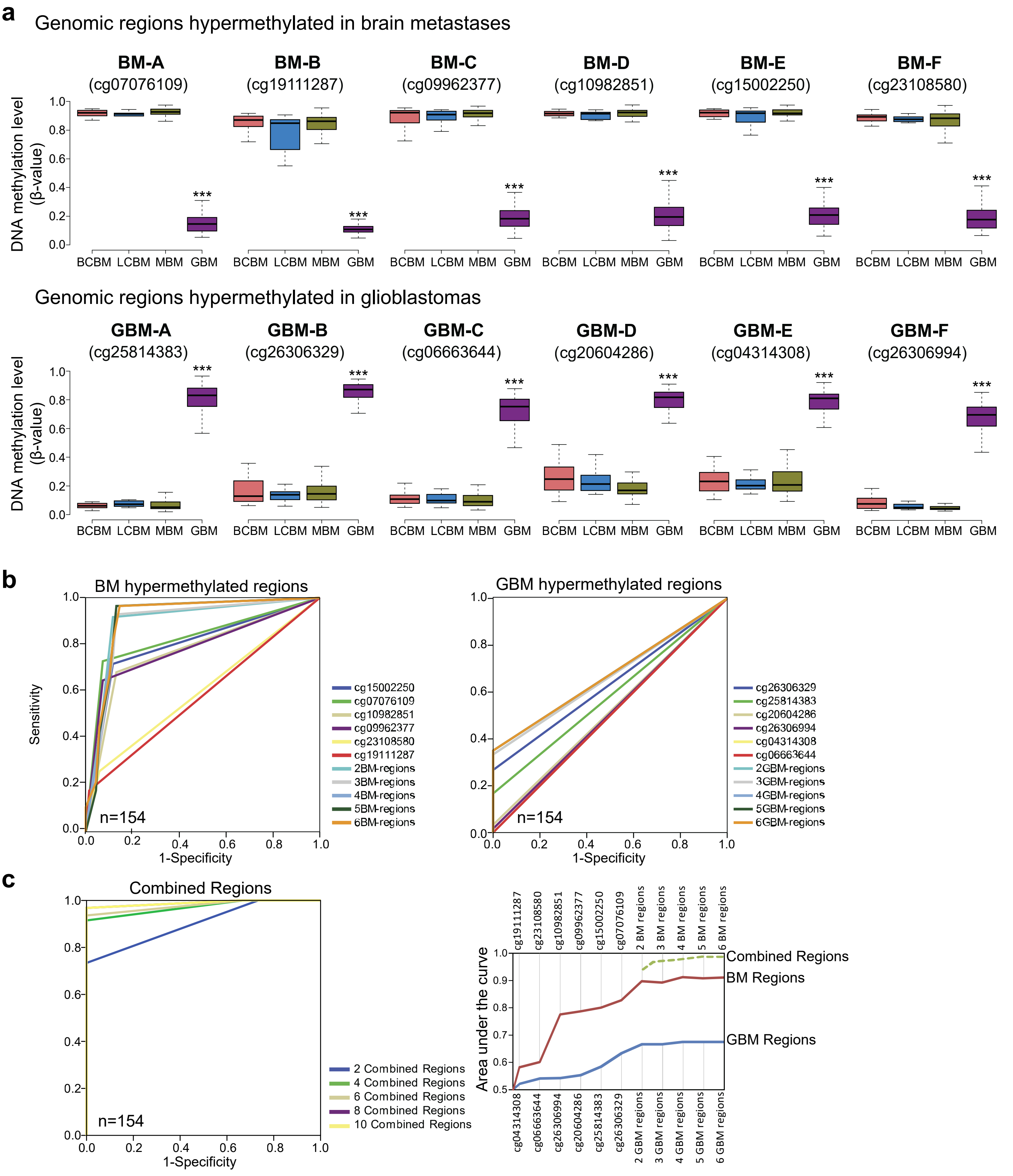


**Supplementary Figure 2**

**Supplementary Figure 2:** a- Boxplots depicting the DNA methylation levels of the 12 genomic regions differentially methylated between primary and metastatic brain tumors (see Supplementary Table 3 for details about the genomic location and distance to nearby genes). The top and bottom of each box represent the first and third quartile, respectively; the internal line represents the median. These regions were selected based on an overall low variance for DNA methylation level within each tumor type, and a large mean DNA methylation (HM450K microarray β-values) difference between primary (GBM, n=60) and metastatic brain tumors (BCBM, n=28; LCBM, n=18; and MBM, n=44). The upper plots include genomic regions consistently hypermethylated in brain metastatic tumors (called herein BM-A to BM-F), and the lower plots include genomic regions consistently hypermethylated in glioblastomas (called herein GBM-A to GBM-F). The differences in the DNA methylation levels between primary and metastatic brain tumors were considered statistically significant (*** Wilcoxon's test; *P*-value < 0.001). b- Receiver operator curves (ROCs) showing the true positive rates (sensitivity) as a function of the false positive rates (1-specificity) for the DNA methylation status of 12 genomic regions, six hypermethylated in metastatic brain tissues (left plot) and six hypermethylated in primary brain tumors (right plot), alone or in combination. In these analyses the DNA methylation levels (β-values) were dichotomized using a β-value cutoff into methylated (β-values ≥0.9) and unmethylated (β-values ≤0.1) statuses (n=154). c- ROCs for combinations of genomic regions distinctly hypermethylated in primary and metastatic brain tumors (n=154; left panel). Area under the curve (AUC) values (y-axis) for the individual and combined genomic regions specifically hypermethylated in GBM and in BM (right panel).

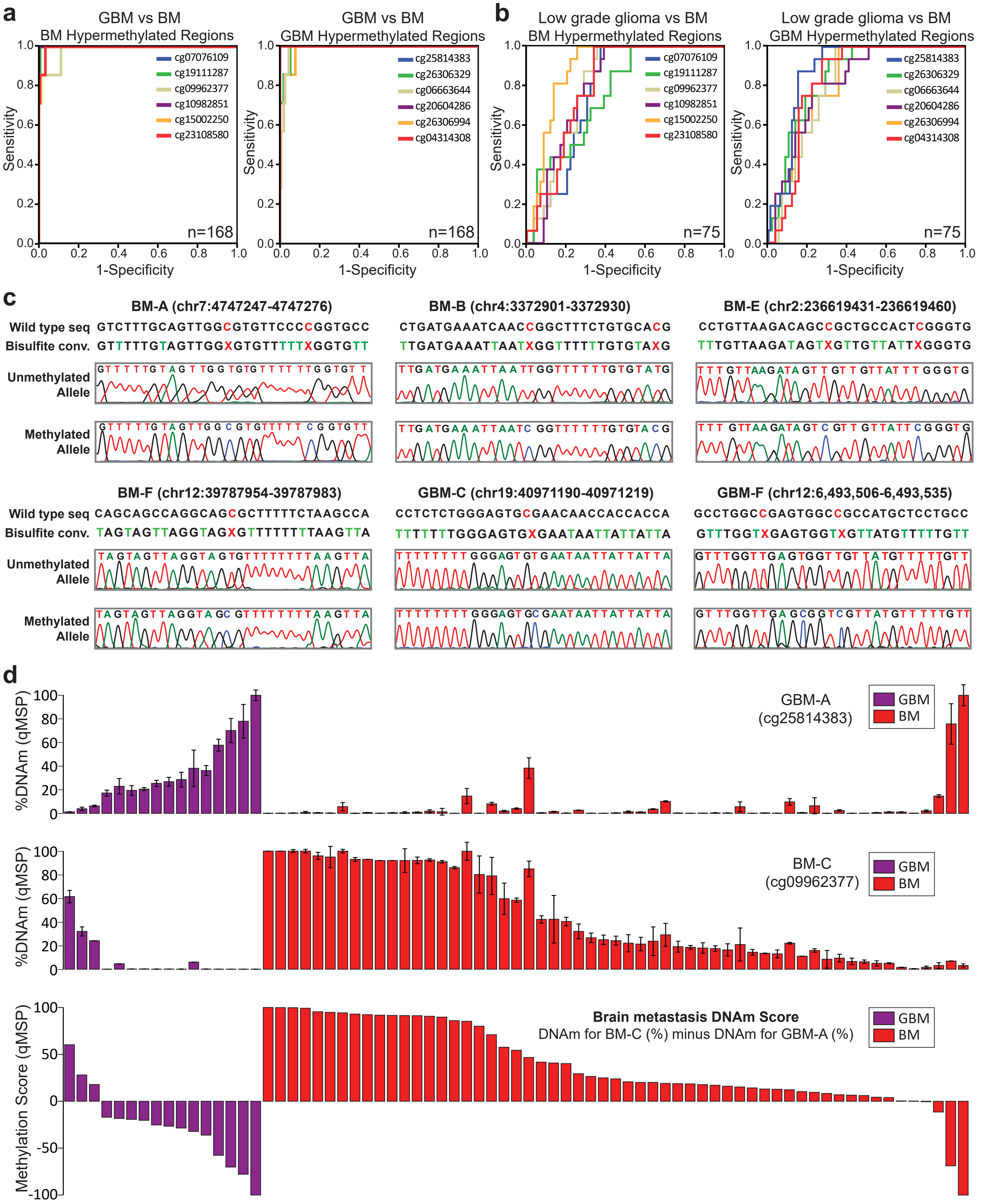


**Supplementary Figure 3**

**Supplementary Figure 3:** a- ROCs distinguishing between GBM (GSE85539) and BM (GSE44661) for genomic regions hypermethylated in metastatic brain tumors (left panel; n=168) and genomic regions hypermethylated in GBM specimens (right panel; n=168). b- ROCs distinguishing between low-grade glioma (LGG; GSE48461) and BM (GSE44661) for genomic regions hypermethylated in metastatic brain tumors (left panel; n=75) and genomic regions hypermethylated in GBM specimens (right panel; n=75). c- Locus-specific bisulfite sequencing for six genomic regions with poor qMSP performance from the *BrainMETH* classifier A (bisulfite sequencing primers sequences for each region can be found in Supplementary Table 3). The cytosines from CpG dinucleotides are highlighted in red in the wild type sequences and replaced by an X in the bisulfite-converted sequences. Cytosines from non-CpG dinucleotides are replaced by a Thymine, highlighted in green. d- qMSP-based DNA methylation level (in percentage) of primary and metastatic brain tumors (n=73) for the most informative GBM-hypermethylated genomic region (GBM-A; upper panel) and the most informative BM-hypermethylated genomic region (BM-C; lower panel). Error bars represent the standard error of the mean (S.E.M.). Brain metastases DNA methylation score generated by subtracting DNA methylation level of a GBM genomic region (GBM-A) from the DNA methylation level of a BM genomic region (BM-C).

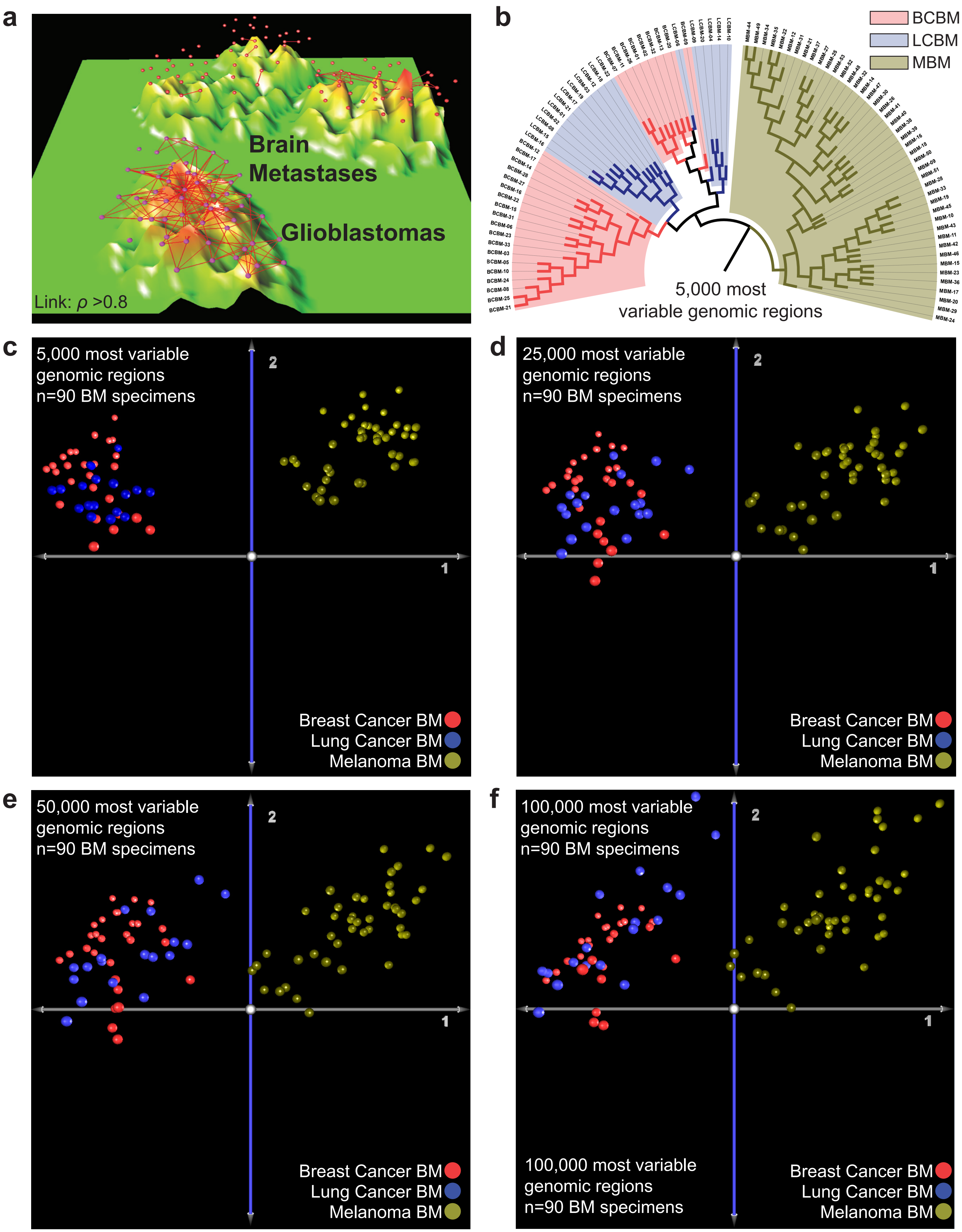


**Supplementary Figure 4**

**Supplementary Figure 4:** a- Three-dimensional terrain map generated with the Spearman's Rank Correlation coefficients (*ρ*) for all brain tumors (n=154) presented in Figure 2a. Red spheres represent metastatic brain tumors (n=94), and purple spheres represent glioblastoma specimens (n=60). Red links indicate brain tumor pairs with a Spearman's *ρ* correlation coefficient >0.8. b- Phylogenetic analysis of all the metastatic brain tumors with confirmed tissue of origin (n=90) using the DNA methylation levels of the top 5,000 most variable genomic regions. The phenetic tree was generated using Euclidean distances among all the metastatic brain tumor tissues. Major branches including brain metastases from the same origin were colored to visualize breast cancer (pink), lung cancer (light blue), and melanoma (brown). c to f- Principal component analyses using the top 5,000 (c), 25,000 (d), 50,000 (e), and 100,000 (f) most variable genomic regions for brain metastasis specimens with confirmed tumor of origin.

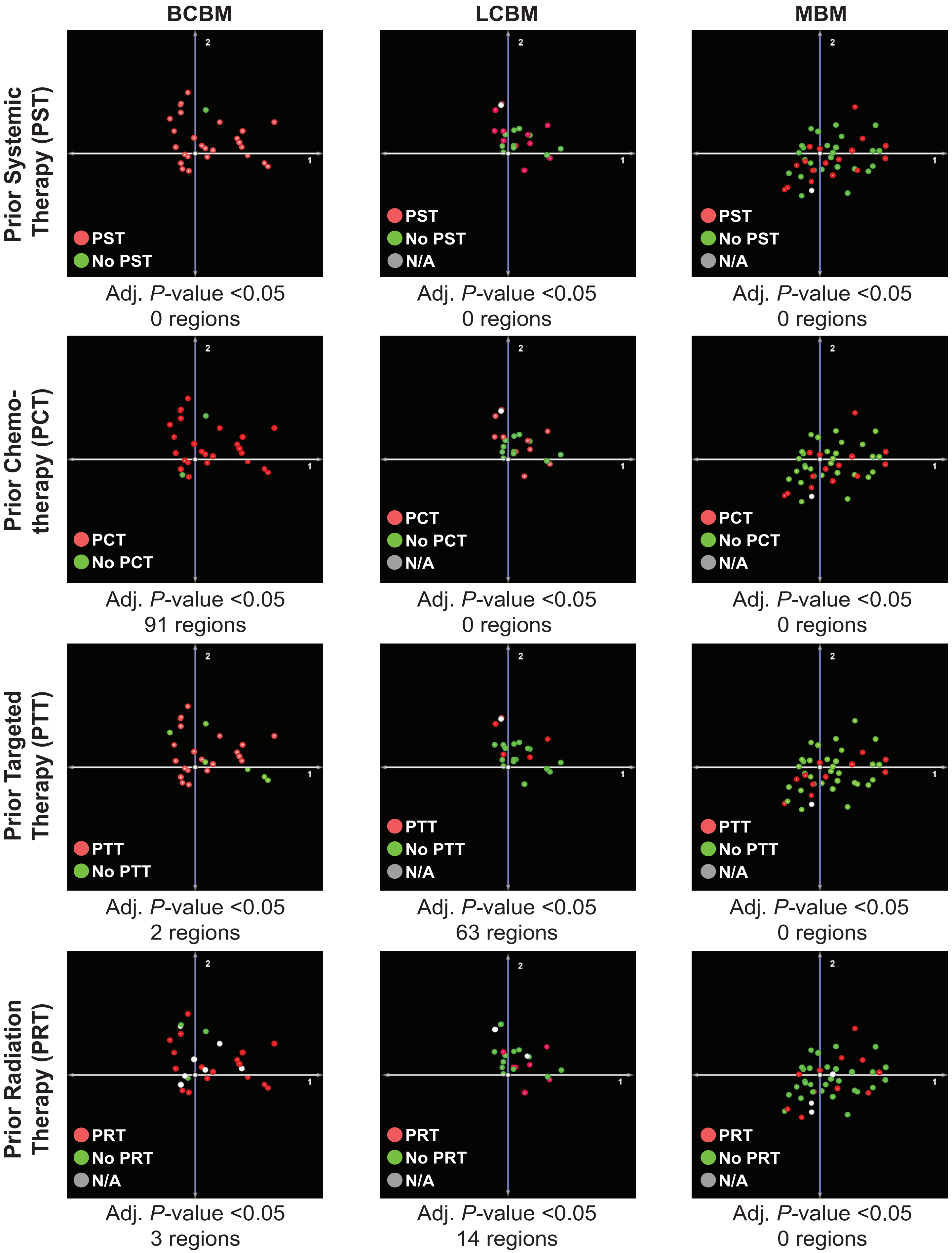


**Supplementary Figure 5**

**Supplementary Figure 5:** Evaluation of potential DNA methylation changes associated with therapeutic interventions administered before obtaining the metastatic brain tumor specimens included in the study (n=94). These multidimensionality scaling analyses were generated using principal component analysis with the DNA methylation levels of 31,818 genomic regions differentially methylated among the brain metastasis types. Due to the variety of therapeutic approaches, each brain metastasis type was evaluated independently for potential influences of four therapeutic modalities as described in Supplementary Table 1. Prior systemic therapies (PST) includes any type of adjuvant or neoadjuvant drug-based therapy for the primary tumor, and also any drug-based therapy for metastatic disease before the metastatic brain tumor specimen was removed. Prior chemotherapy (PCT) includes the use of any type of chemotherapeutic agent to treat the primary tumor, extracranial metastases, and/or brain metastases preceding specimen removal. Prior targeted therapy (PTT) includes the use of any type of targeted agent in the neoadjuvant, adjuvant and/or metastatic settings. In this category, we identified the use of anti-HER2 therapy, endocrine therapy, anti-EGFR therapy, BRAF inhibitors, immunotherapy, or other investigational targeted drugs, such as trastuzumab, lapatinib, tamoxifen, aromatase inhibitors, erlotinib, afatinib, dabrafenib, vemurafenib, ipilimumab, pembrolizumab or nivolumab. Prior radiation therapy (PRT) includes those cases in which radiation therapy was used for the loco-regional treatment of the primary tumor, extracranial metastases, and/or the use of stereotactic radiosurgery or whole brain radiotherapy before the brain metastasis was removed. In addition to the multidimensional scaling, the number of differentially methylated genomic regions are presented for each treatment modality (Wilcoxon test; Adjusted *P*-value <0.05).

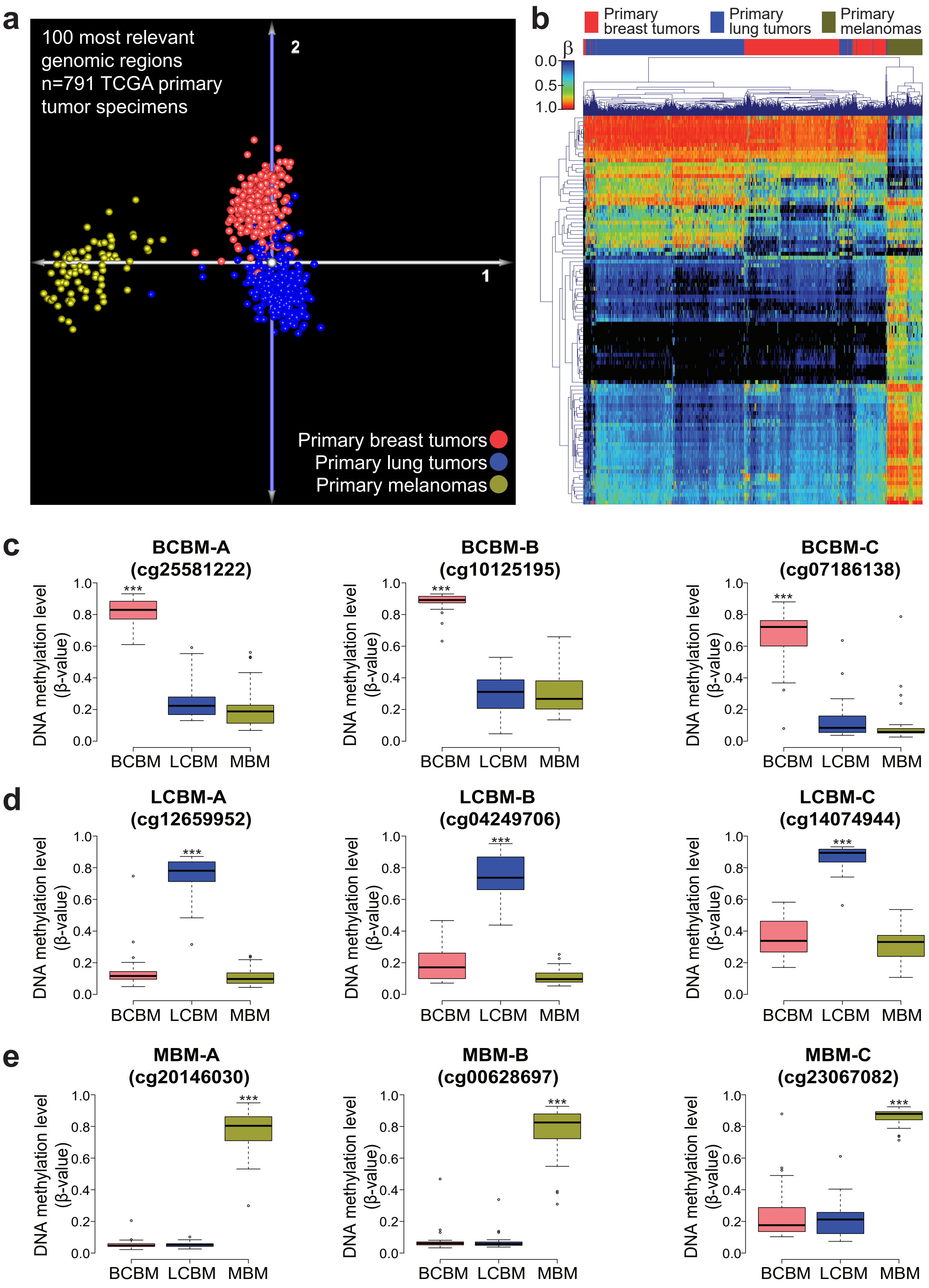


**Supplementary Figure 6**

**Supplementary Figure 6:** a- Principal component analysis for primary tumors from breast (n=401), lung (n=307), and melanoma (n=83) using the top 100 most informative genomic regions for the classification of metastatic brain tumors. Only TCGA primary tumor specimens detection *P*-value greater than 0.01 for the 100 selected CpG sites were included in this analysis. b- Unsupervised hierarchical cluster analysis for TCGA primary tumor specimens using the top 100 most informative for the classification of metastatic brain tumors. c to e- Boxplots showing the DNAm levels (HM450K microarray β-values) of nine genomic regions differentially methylated among the three types of brain metastases (***Wilcoxon test; P-value <0.001; n=94; see Supplementary Table 6 for details about the genomic location and distance to nearby genes). This set of regions includes three CpG sites hypermethylated in BCBM (c), three CpG sites hypermethylated in LCBM (d), and three CpG sites hypermethylated in MBM (e). The top and bottom of each box represent the first and third quartile, respectively; the internal line represents the median.

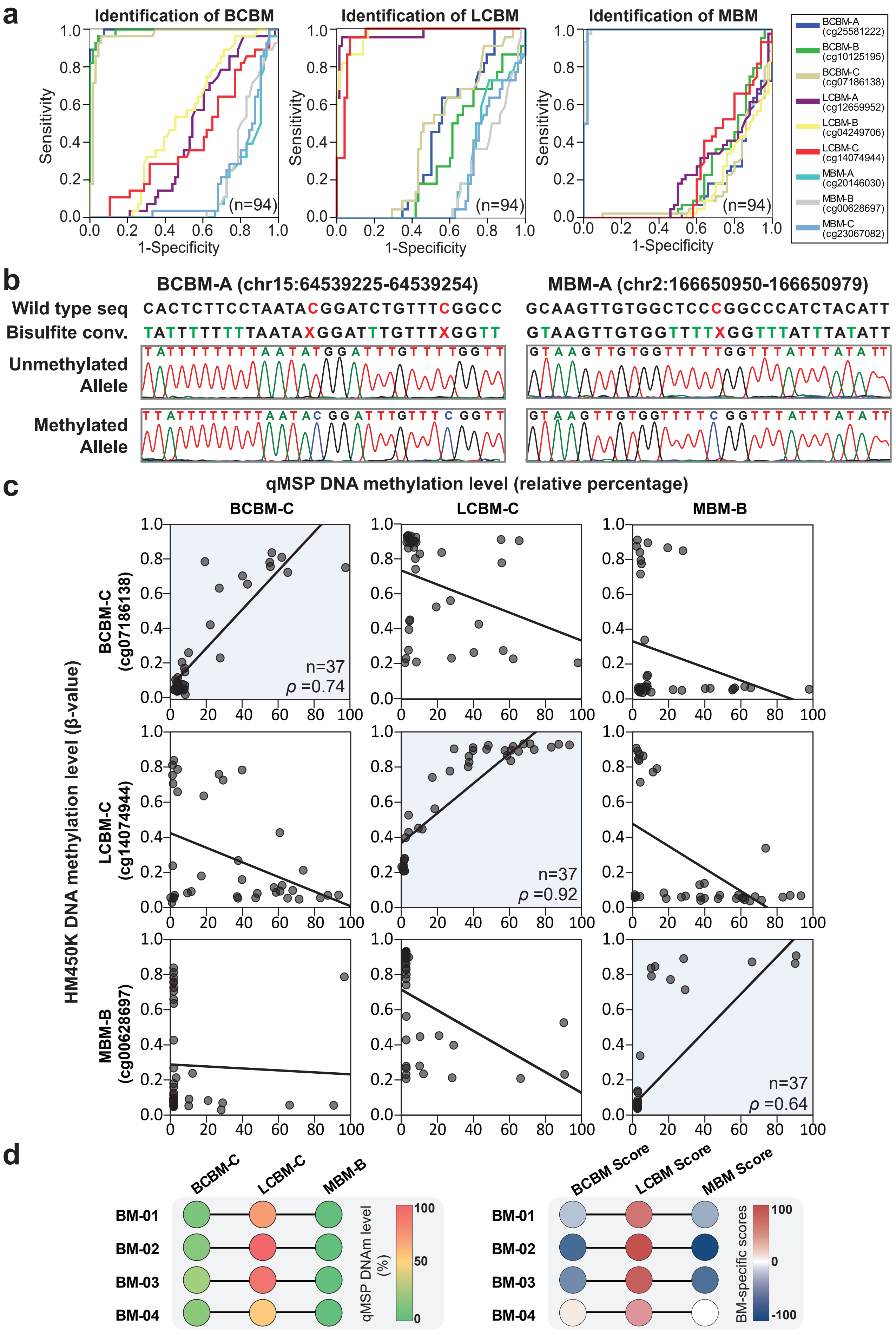


**Supplementary Figure 7**

**Supplementary Figure 7:** a- ROCs distinguishing between the three types of brain metastases (n=94) using the DNA methylation levels (HM450K microarray β values) of the nine differentially methylated genomic regions (Supplementary Table 6). b- Locus-specific bisulfite sequencing for genomic regions with poor qMSP performance from the brain metastasis tumor of origin classifier (n=2; bisulfite sequencing primers sequences for each region can be found in Supplementary Table 6). The Cytosines from CpG dinucleotides are highlighted in red in the wild type sequences and replaced by an X in the bisulfite-converted sequences. Cytosines from non-CpG dinucleotides are replaced by a Thymine, highlighted in green. c- Spearman´s *ρ* correlation among DNA methylation levels assessed by HM450K (β-values, y-axes) and targeted approach (qMSP; x-axes) for the selected three genomic regions (n=37). d- qMSP analysis of the three selected genomic regions of the four samples with an uncertain primary origin of BM. The left panel shows the DNAm level (percentage) for each region. The right panel shows the DNAm scores specific for each type of BM. Each score was calculated as follow: BCBMscore = DNAm level of BCBM-C minus DNAm level of LCBM-C minus DNAm level of MBM-B; LCBMscore = DNAm level of LCBM-C minus DNAm level of BCBM-C minus DNAm level of MBM-B; and MBMscore = DNAm level of MBM-B minus DNAm level of LCBM-C minus DNAm level of BCBM-C.

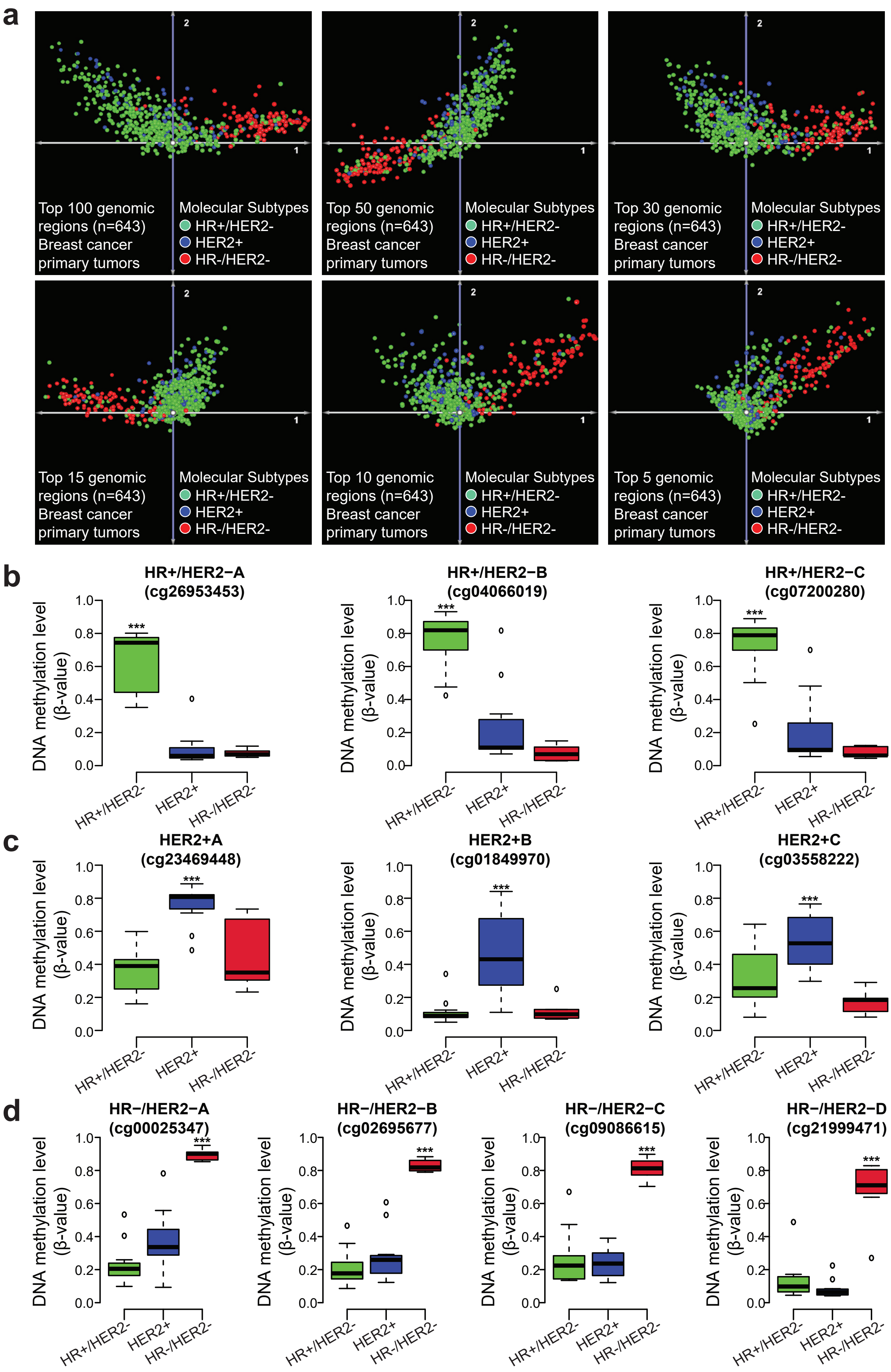


**Supplementary Figure 8**

**Supplementary Figure 8:** a- Principal component analyses for primary breast cancer using the top 100, 50, 30, 15, 10, and 5 most informative genomic regions for the classification of breast cancer subtypes. Only TCGA primary breast tumor specimens detection *P*-value greater than 0.01 for the 100 selected CpG sites were included in this analysis (n=643). b to d- Boxplots showing the DNAm levels (HM450K microarray β-values) of 10 genomic regions differentially methylated among the three breast cancer brain metastases molecular subtypes (n=24; see Supplementary Table 10 for details about the genomic location and distance to nearby genes). This set of regions includes three CpG sites hypermethylated in HR+ and HER2- breast cancer brain metastasis specimens (b), three CpG sites hypermethylated in HER2+ breast cancer brain metastasis specimens (c), and four CpG sites hypermethylated in HR- and HER2- breast cancer brain metastasis specimens (d). The top and bottom of each box represent the first and third quartile, respectively; the internal line represents the median. ***Wilcoxon test; *P*-value <0.001;

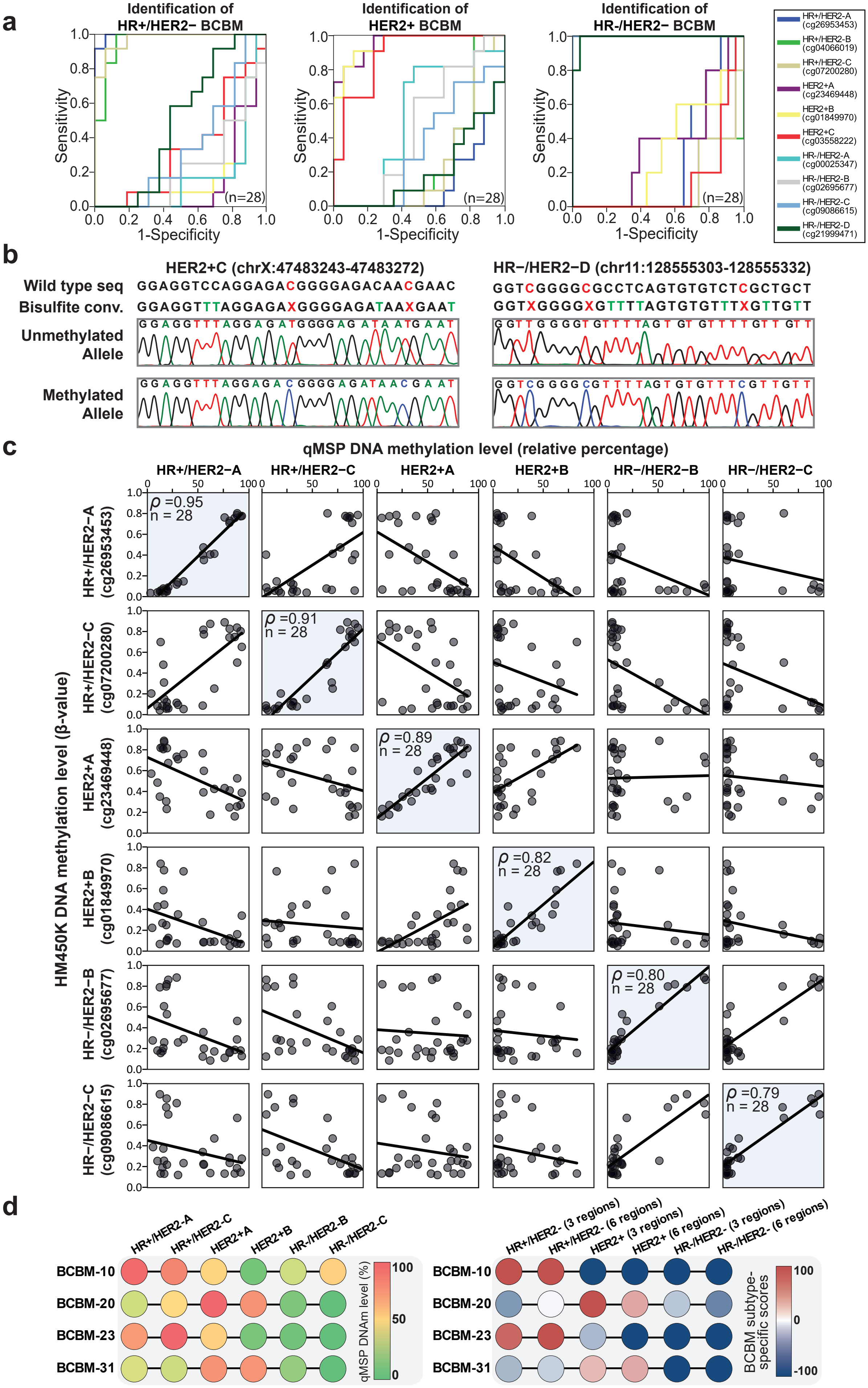


Supplementary Figure 9

**Supplementary Figure 9:** a- ROCs distinguishing among the three breast cancer brain metastasis molecular subtypes using the DNA methylation levels (HM450K microarray β-values; n=28) of the 10 differentially methylated regions (Supplementary Table 8). b- Locus-specific bisulfite sequencing for the genomic regions with poor qMSP performance from the breast cancer brain metastasis molecular subtype classifier (n=2; bisulfite sequencing primers sequences for each region can be found in Supplementary Tables 10). The Cytosines from CpG dinucleotides are highlighted in red in the wild type sequences and replaced by an X in the bisulfite-converted sequences. Cytosines from non-CpG dinucleotides are replaced by a Thymine, highlighted in green. c- Spearman´s $\rho$ correlation among DNA methylation levels assessed by HM450K (β-values, y-axes) and targeted approach (qMSP; x-axes) for the selected three genomic regions (n=28). d- qMSP analysis of the selected six genomic regions of the four BCBM samples without IHC assessment of ER, PgR, and HER2 at the moment of initial diagnosis. Left panel shows the DNAm level (percentage) for each region. Right panel shows the DNAm scores specific to each breast cancer molecular subtypes considering three (one per molecular subtype) or six (two per molecular subtypes) genomic regions. BCBM subtype-specific scores for the combination of three genomic regions were calculated as follow: HR+/HER2-scores = DNAm level of HR+/HER2- minus DNAm level of HER2+ minus DNAm level of HR-/HER2; HER2+scores = DNAm level of HER2+ minus DNAm level of HR+/HER2- minus DNAm level of HR-/HER2; and HR-/HER2-scores = DNAm level of HR-/HER2- minus DNAm level of HR+/HER2- minus DNAm level of HER2+. For the combination of six genomic regions, the average of regions specific of each BCBM subtype were considered.

**Supplementary Note 1:** Clinical and demographic information for all the patients with brain metastasis included in the study.

---

**Breast cancer brain metastasis (BCBM) specimens**

**Specimen ID:** BCBM-01

Female patient with a diagnosis of invasive breast cancer at 37 years old. The patient developed a single cerebellar brain metastasis followed by lung and liver metastases. The immunohistochemistry markers of the brain metastasis were ER negative, PgR negative and HER2 positive.

**Specimen ID:** BCBM-02

Female patient with a diagnosis of occult breast cancer with liver metastasis at 50 years old. Four years later, the patient developed multiple cerebellar and frontal brain metastases. The immunohistochemistry markers of the brain metastases were ER negative, PgR negative and HER2 positive.

**Specimens ID:** BCBM-03 and BCBM-04

Female patient with a diagnosis of triple negative breast cancer at 34 years old. The patient initially developed lung and bone metastases, followed by 2 synchronous brain metastases in the frontal and parietal lobes, at 52 years old. The immunohistochemistry markers of the frontal lesion were ER negative, PgR negative and HER2 negative.

**Specimens ID:** BCBM-05 and BCBM-19

Female patient with a diagnosis of invasive breast cancer at 50 years old. Two years later, the patient developed a frontal brain metastasis, followed by a subsequent parietal brain metastasis 3 months later. The immunohistochemistry markers of the first brain metastasis were ER negative, PgR negative and HER2 positive.

**Specimen ID:** BCBM-06

Female patient with a diagnosis of invasive breast cancer at 47 years old. The patient presented bone and liver metastases, followed by lung metastases. She finally developed a single parietal-occipital brain metastasis. The immunohistochemistry markers of the brain metastasis were ER positive, PgR positive and HER2 positive.

**Specimen ID:** BCBM-07

Female patient with a diagnosis of invasive ductal breast cancer, stage IA, at 76 years old. The patient presented a single cerebellar metastasis 4 years after the diagnosis of the primary breast cancer. The immunohistochemistry markers of the brain metastasis were ER negative, PgR negative and HER2 negative.

**Specimen ID:** BCBM-08

Female patient with a diagnosis of invasive ductal breast cancer, stage IA, at 40 years old. The patient initially developed lung and bone metastases, followed by a single parietal brain metastasis at 50 years old. The immunohistochemistry markers of the brain metastasis were ER positive, PgR positive and HER2 positive.

**Specimen ID:** BCBM-09

Female patient with a diagnosis of triple negative breast cancer, at 24 years old. The patient initially developed poorly differentiated metastasis at the chest wall, mediastinum lymph nodes and bone, followed by multiple brain metastases. The immunohistochemistry markers of the brain metastases were ER negative, PgR negative and HER2 negative.

**Specimen ID:** BCBM-10

Female patient with a diagnosis of occult breast cancer with bone metastasis at 75 years old. Two years later, she developed a single brain metastasis at temporal lobe. The immunohistochemistry markers of the brain metastasis were retrospectively examined during this study and were ER positive, PgR positive and HER2 negative, in concordance with the immunohistochemistry profile of the previous bone metastasis.

**Specimen ID:** BCBM-11

Female patient with a diagnosis of invasive ductal carcinoma triple negative breast cancer, stage IIA, at 38 years old. The patient initially developed lung and liver metastases, followed by multiple brain metastases at 40 years old. The immunohistochemistry markers of the brain metastases were ER negative, PgR negative and HER2 negative.

**Specimen ID:** BCBM-12

Female patient with a diagnosis of invasive breast cancer, at 48 years old. The patient initially developed metastasis in the mediastinum, followed by multiple brain metastases at 50 years old. The immunohistochemistry markers of the brain metastases were ER positive, PgR negative and HER2 positive.

**Specimen ID:** BCBM-13

Female patient with a diagnosis of inflammatory breast cancer, stage IIIB, at 50 years old. The patient developed a single brain metastasis in cerebellum 6 years after the diagnosis of the primary breast cancer. The immunohistochemistry markers of the brain metastasis were ER negative, PgR negative and HER2 negative.

**Specimen ID:** BCBM-14

Female patient with a diagnosis of invasive breast cancer, at 39 years old. The patient initially developed bone, lung and liver metastases, followed by multiple brain metastases at 44 years old. The immunohistochemistry markers of the brain metastases were ER positive, PgR negative and HER2 negative.

**Specimen ID:** BCBM-15

Female patient with a diagnosis of mucinous breast cancer, stage IIA, at 60 years old. The patient developed bone metastases followed by a single brain metastasis in temporal lobe 5 years after the diagnosis of the primary breast cancer. The immunohistochemistry markers of the brain metastasis were ER positive, PgR positive and HER2 negative.

**Specimen ID:** BCBM-16

Female patient with a diagnosis of occult breast cancer with liver and cerebellum metastases at 73 years old. The immunohistochemistry markers of the brain metastases were ER positive, PgR positive and HER2 negative.

**Specimen ID:** BCBM-17

Female patient with a diagnosis of invasive breast cancer, stage IIIC, at 37 years old. The patient developed parietal brain metastases 3 years after the diagnosis of the primary breast cancer. The immunohistochemistry markers of the brain metastases were ER positive, PgR negative and HER2 negative.

**Specimen ID:** BCBM-20

Female patient with a diagnosis of *de novo* stage IV breast cancer, due to the presence of bone, lung and liver metastases, at 55 years old. Two years later, she developed a single cerebellum metastasis. The immunohistochemistry markers were retrospectively examined during this study and were ER negative, PgR negative and HER2 positive.

**Specimen ID:** BCBM-21

Female patient with a diagnosis of invasive lobular breast cancer, stage IIA, at 43 years old. The patient developed frontal and temporal brain metastases 3 years after the diagnosis of the primary breast cancer. The immunohistochemistry markers of the brain metastases were ER positive, PgR positive and HER2 negative.

**Specimen ID:** BCBM-22

Female patient with a diagnosis of invasive breast cancer, stage IIB, at 58 years old. The patient developed lymph node, bone and liver metastases and subsequently presented multiple brain metastases at 70 years old. The immunohistochemistry markers of the brain metastases were ER positive, PgR negative and HER2 negative.

**Specimen ID:** BCBM-23

Female patient with a diagnosis of invasive ductal breast cancer, stage IIIA, at 36 years old. Five years later, she developed metastatic disease at mediastinum, bone, and lung. At the age of 46 years, she presented multiple cerebellum metastases. The immunohistochemistry markers were retrospectively examined during this study and were ER positive, PgR negative and HER2 negative, similar to the immunohistochemistry profile of the primary breast cancer.

**Specimen ID:** BCBM-24

Female patient with a diagnosis of invasive ductal breast cancer, stage IB, at 59 years old. Two years later, she presented a chest wall recurrence. At the age of 71 years, she presented lung metastases followed by multiple brain metastases at frontal and occipital lobes. The immunohistochemistry markers of the brain metastases were ER positive, PgR positive and HER2 positive.

**Specimen ID:** BCBM-25

Female patient with a diagnosis of occult breast cancer with bone and lung metastases at 55 years old. The patient developed multiple brain metastases at 67 years old. The immunohistochemistry markers of the brain metastases were ER positive, PgR positive and HER2 negative.

**Specimen ID:** BCBM-26

Female patient with a diagnosis of invasive breast cancer, at 54 years old. Subsequently, she developed a single brain metastasis in the thalamus 4 years after the diagnosis of the primary

breast cancer. The immunohistochemistry markers of the brain metastasis were ER negative, PgR negative and HER2 negative.

**Specimen ID:** BCBM-27

Female patient with a diagnosis of brain metastasis due to breast cancer, at 61 years old. The immunohistochemistry markers of the brain metastasis were ER positive, PgR positive and HER2 negative.

**Specimen ID:** BCBM-28

Female patient with a diagnosis of brain metastasis due to breast cancer, at 65 years old. The immunohistochemistry markers of the brain metastasis were ER positive, PgR positive and HER2 negative.

**Specimen ID:** BCBM-31

Female patient with a diagnosis of invasive breast cancer, at 33 years old. Two years later, she developed a brain metastasis. The immunohistochemistry markers were retrospectively examined during this study and were ER positive, PgR positive and HER2 positive.

**Specimen ID:** BCBM-32

Female patient with a diagnosis of brain metastasis due to breast cancer, at 45 years old. The immunohistochemistry markers of the brain metastasis were ER positive, PgR positive and HER2 positive.

**Specimen ID:** BCBM-33

Female patient with a diagnosis of invasive ductal breast cancer, stage IIB, at 38 years old. Two years later, she presented cerebellum metastases. The immunohistochemistry markers of the brain metastases were ER negative, PgR negative and HER2 positive.

**Lung cancer brain metastasis (LCBM) specimens**

**Specimen ID:** LCBM-01

Female patient with a diagnosis of non-small cell lung cancer at 59 years old. The patient developed brain metastasis at 62 years old, similar to the previous bronchial biopsy.

**Specimen ID:** LCBM-02

Female patient with a diagnosis of non-small cell lung cancer at 63 years old. The patient developed brain metastasis at 65 years old, similar to the previous right lower lobe lung tumor. Immunohistochemistry markers were TTF-1 positive, Napsin-A positive.

**Specimen ID:** LCBM-03

Female patient with a diagnosis of non-small cell lung cancer with adenosquamous histology at 74 years old. Six months later, she developed brain metastasis similar to lung cancer with adenosquamous histology. Immunohistochemistry markers were TTF-1 positive, Napsin-A positive.

**Specimen ID:** LCBM-04

Female patient with a history of non-small cell lung cancer. The patient developed brain metastasis at 71 years old, compatible with pulmonary adenocarcinoma metastasis with neuroendocrine differentiation.

**Specimen ID:** LCBM-06

Female patient with a history of non-small cell lung cancer. The patient developed brain metastasis at 67 years old, compatible with metastatic adenocarcinoma of lung/bronchogenic origin. The brain metastasis was KRAS and EGFR wild-type.

**Specimen ID:** LCBM-08

Female patient with a history of non-small cell lung cancer at 66 years old. The patient developed brain metastasis 5 months later, with identical immunoprofile to primary lung carcinoma.

**Specimen ID:** LCBM-09

Female patient with a history of undifferentiated non-small cell lung cancer, presenting brain metastasis of similar histology at 66 years old.

**Specimen ID:** LCBM-10

Female patient with a diagnosis of brain metastasis from small cell lung cancer, at 71 years old. Immunohistochemistry markers were TTF-1 negative, synaptophysin positive, CK7 positive/CK20 negative.

**Specimen ID:** LCBM-12

Female patient with a diagnosis of non-small cell lung cancer with squamous histology at 86 years old. The patient developed brain metastasis 7 months later, with identical immunoprofile to primary lung carcinoma.

**Specimen ID:** LCBM-14

Male patient with a diagnosis of brain metastasis from small cell lung cancer, at 67 years old. Immunohistochemistry markers were TTF-1 positive, synaptophysin positive and chromogranin positive.

**Specimen ID:** LCBM-15

Male patient with a diagnosis of brain metastasis from non-small cell lung cancer with adenocarcinoma histology, at 88 years old. Immunohistochemistry markers were TTF-1 positive, napsin-A positive, and CK7 positive/CK20 negative.

**Specimen ID:** LCBM-16

Female patient with a diagnosis of brain metastasis from non-small cell lung cancer with adenocarcinoma histology, at 69 years old. Immunohistochemistry markers were TTF-1 positive, napsin-A positive, and CK7 positive/CK20 negative.

**Specimen ID:** LCBM-17

Female patient with a diagnosis of brain metastasis from non-small cell lung cancer with adenocarcinoma histology, at 64 years old.

**Specimen ID:** LCBM-18

Male patient with a diagnosis of brain metastasis of unknown primary at 61 years old. The patient has a history of skin basal cell carcinoma and melanoma in situ. The immunohistochemistry markers were compatible with pulmonary metastatic adenocarcinoma.

**Specimen ID:** LCBM-19

Female patient with a diagnosis of non-small cell lung cancer with adenocarcinoma histology at 81 years old. The patient developed brain metastasis 2 years later, with similar immunoprofile to primary lung carcinoma.

**Specimen ID:** LCBM-20

43 years old male patient with a history of an apical lung mass, presenting a single brain metastasis with histology of high-grade neuroendocrine carcinoma, synaptophysin positive, chromogranin positive and CK7 positive.

**Specimen ID:** LCBM-21

76 years old female patient with a diagnosis of brain metastasis compatible with lung adenocarcinoma origin. Immunohistochemistry markers were TTF-1 positive, napsin-A positive, and CK7 positive/CK20 negative. The patient has a history of a lung mass, heavier smoker. The lung biopsy couldn't be performed due to the patient's poor health condition.

**Specimen ID:** LCBM-22

Female patient with a diagnosis of brain metastasis from non-small cell lung cancer with adenocarcinoma histology, at 68 years old.

**Melanoma brain metastasis (MBM) specimens**

**Specimen ID:** MBM-09

Male patient diagnosed with stage IIB melanoma on the scalp at 60 years old. The patient developed scalp recurrence and regional lymph node metastasis, followed by lung metastasis. Finally, presented a single sellar metastasis, NRAS mutated, 73 months after the diagnosis of the primary tumor. The patient was deceased 25 months after the brain metastasis diagnosis.

**Specimen ID:** MBM-10

Female patient diagnosed with stage IIIB melanoma in the trunk at 51 years old. The patient developed cutaneous metastasis, and finally, presented a single brain metastasis at the right basal ganglia, BRAF and NRAS wild-type, 12 years after the diagnosis of the primary tumor. The patient was deceased 43 months after the brain metastasis diagnosis.

**Specimen ID:** MBM-11

Male patient diagnosed with occult melanoma at 80 years old with simultaneous lung and brain metastasis at initial diagnosis. He presented multiple brain metastases at frontal, temporal lobes and cerebellum. The patient was deceased 6 months after the brain metastasis diagnosis.

**Specimen ID:** MBM-12

Male patient diagnosed with occult melanoma at 43 years old, presenting 2 brain metastases at the left parietal and left temporal-parietal lobes, NRAS mutated. The patient was deceased 24 months after the brain metastasis diagnosis.

**Specimen ID:** MBM-14

Male patient, with a diagnosis of nodular melanoma on the scalp stage IIIC at 60 years-old. He subsequently developed cutaneous metastasis, and finally, presented a single brain metastasis at the left frontal lobe, BRAF mutated, 20 months after the diagnosis of the primary tumor. The patient was deceased 11 months after the brain metastasis diagnosis.

**Specimen ID:** MBM-15

Female patient, with a diagnosis of melanoma on the trunk at 44 years-old who presented 2 brain metastases at the left frontal lobe, NRAS mutated, 11 months after the diagnosis of the primary tumor. The patient is alive with persistent disease 5.6 years after the brain metastasis diagnosis.

**Specimen ID:** MBM-16

Female patient, with a diagnosis of melanoma at 44 years-old who presented a single brain metastasis in the right frontal lobe, BRAF mutated, 4 months after the initial diagnosis. The patient is alive with persistent disease 3 years after the brain metastasis diagnosis.

**Specimen ID:** MBM-17

Female patient, with a diagnosis of superficial spreading melanoma on the trunk who presented a single brain metastasis at the left parietal lobe, BRAF mutated, 21.6 years after the initial diagnosis. The patient was deceased 11 months after the brain metastasis diagnosis.

**Specimen ID:** MBM-18

75 years old Female patient, diagnosed with stage IIB nodular melanoma in the calf. The patient presented a single brain metastasis at the left frontal lobe, NRAS mutated, 10 years after primary diagnosis. The patient was deceased 5 months after the brain metastasis diagnosis.

**Specimen ID:** MBM-19

Male patient, diagnosed with melanoma on the face, stage IB at 54 years-old. The patient subsequently developed cutaneous and regional lymph nodes metastasis, followed by liver metastasis. Finally, presented 2 brain metastases at the left Sylvian fissure, BRAF mutated, 22 months after the diagnosis of the primary tumor. The patient died 17 months after the brain metastasis diagnosis.

**Specimen ID:** MBM-20

Female patient, with a diagnosis of desmoplastic melanoma on the upper arm stage IIB at 62 years-old. The patient developed lung metastasis and 8 years and 10 months after the diagnosis of the primary melanoma. The patient presented a single brain metastasis in the right frontal lobe, BRAF/NRAS wild type. The patient is alive with persistent disease 16 months after the brain metastasis diagnosis.

**Specimen ID:** MBM-21

Female patient, with a diagnosis of melanoma at 56 years-old, stage IIIC. The patient presented a single brain metastasis at the left parietal lobe, BRAF/NRAS wild-type, 30 months after the diagnosis of the primary tumor. The patient died 3 years after the diagnosis of brain metastasis.

**Specimen ID:** MBM-22

Male patient, with a diagnosis of nodular melanoma on the face stage IIA at 48 years old. The patient presented a single brain metastasis at the right temporal region, NRAS mutated, 52 months after the diagnosis of the primary tumor. He previously developed bone and lung metastases. The patient died 3 years after the brain metastasis diagnosis from a non-caner related death.

**Specimen ID:** MBM-23

Male patient, with a diagnosis of melanoma on the shoulder, stage IIB, at 70 years old. The patient subsequently developed right posterior chest cutaneous metastasis, and finally, presented a single brain metastasis at the right frontal lobe, NRAS mutated, 28 months after the diagnosis of the primary tumor. The patient was deceased 11 months after the brain metastasis diagnosis.

**Specimen ID:** MBM-24

Male patient, with a diagnosis of melanoma at 65 years-old, stage II. The patient subsequently developed liver, lung and bone metastases, and finally, presented a single brain metastasis at posterior left inferior gyrus lesion, BRAF/NRAS wild-type, 6 years after the diagnosis of the primary tumor. The patient was deceased 4 months after the brain metastasis diagnosis.

**Specimen ID:** MBM-25

Male patient, with a diagnosis of stage IIA thoracic melanoma at 60 years-old who subsequently developed 2 brain metastases at right upper parietal, BRAF mutated, 23 months after the diagnosis of the primary tumor. The patient was deceased 7.8 years after the brain metastasis diagnosis.

**Specimen ID:** MBM-26

Male patient, with a diagnosis of melanoma on the flank stage IIA at 69 years-old. The patient then developed lung metastasis, and finally, presented a single brain metastasis at right temporal, NRAS mutated, 5 years after the diagnosis of the primary tumor. The patient was deceased 7 months after the brain metastasis diagnosis.

**Specimen ID:** MBM-27

71-year-old male patient diagnosed with stage IIA melanoma on the shoulder. The patient subsequently developed bone metastasis, and finally, presented a single brain metastasis in the left temporal-parietal region, BRAF mutated, 11 years after the diagnosis of the primary tumor. The patient was deceased 6 months after the brain metastasis diagnosis.

**Specimen ID:** MBM-28

Male patient, with a thoracic nodular melanoma stage IIC at 44 years-old. The patient presented a single brain metastasis at right parietal-occipital, BRAF mutated, 31 months after the diagnosis of the primary tumor. The patient is alive and disease-free 8 years after the brain metastasis diagnosis.

**Specimen ID:** MBM-29

Male patient, diagnosed with stage IIA melanoma on the forehead, at 74 years-old. The patient presented a single brain metastasis at the right occipital lobe, NRAS mutated. The patient was deceased 9 months after the brain metastasis diagnosis.

**Specimen ID:** MBM-30

Male patient diagnosed with stage IIB thoracic desmoplastic melanoma at 61 years-old. The patient presented 2 brain metastases in the right occipital lobe, BRAF/NRAS wild-type, 38 months after the diagnosis of the primary tumor. The patient is alive with persistent disease 5 months after the brain metastasis diagnosis.

**Specimen ID:** MBM-31

Male patient diagnosed with stage IIIC melanoma on the trunk at 65 years-old. The patient subsequently developed lung and regional lymph node metastasis, and finally, presented 2 brain metastases at the left frontal lobe, NRAS mutated, 6 years after the diagnosis of the primary tumor. The patient was deceased 3 months after the brain metastasis diagnosis.

**Specimen ID:** MBM-32

42-year-old female patient, with a diagnosis of stage IB superficial spreading melanoma on the trunk. The patient subsequently developed right neck lymph node metastasis, and finally, presented a single brain metastasis at the left temporal lobe, BRAF mutated, 58 months after the diagnosis of the primary tumor. The patient was deceased 3.9 years after the brain metastasis diagnosis.

**Specimen ID:** MBM-33

Male patient, with a diagnosis of stage IIB nodular melanoma on the trunk at 60 years-old. The patient subsequently developed cutaneous metastasis followed by liver metastasis. Finally, presenting a single brain metastasis at the left frontal lobe, BRAF mutated, 20 months after the diagnosis of the primary tumor. The patient was deceased 11 months after the brain metastasis diagnosis.

**Specimen ID:** MBM-34

Male patient diagnosed with stage IIA superficial spreading melanoma on the head/neck at 52 years old, who presented two brain metastases at right parietal-occipital, NRAS mutated 34 months after primary diagnosis. The patient was deceased 3 months after brain metastases diagnosis.

**Specimen ID:** MBM-35

Male patient, with a diagnosis of stage IIB nodular melanoma on the scalp at 61 years old. The patient subsequently developed cutaneous metastasis followed by lymph node metastasis. Finally presented a single brain metastasis at the frontal cortex, BRAF mutated 17 months after the primary diagnosis. The patient was deceased 15 months after brain metastasis diagnosis.

**Specimen ID:** MBM-36

27-year-old male patient diagnosed with stage IB thoracic melanoma. The patient then developed right neck lymph node metastasis. Finally presented multiple brain metastases at the right and left frontal cortex, BRAF mutated 12 years after the primary diagnosis. The patient is alive with persistent disease 6 years after brain metastases diagnosis.

**Specimen ID:** MBM-37

56-year-old male patient diagnosed with melanoma in situ on extremities. The patient subsequently presented right inguinal metastasis and finally developed a single brain metastasis in the left frontal lobe, NRAS mutated 2 years after the primary diagnosis. The patient died 22 months after brain metastasis diagnosis.

**Specimen ID:** MBM-38

Female patient with a diagnosis of stage IB superficial spreading melanoma on the shoulder at 38 years old. The patient subsequently developed right neck lymph node metastasis. Eleven years after the primary diagnosis she presented a single brain metastasis at the right frontal cortex, BRAF mutated. The patient is alive with persistent disease 7.2 years after the brain metastasis diagnosis.

**Specimen ID:** MBM-39

61-year-old male patient diagnosed with stage IIA superficial spreading melanoma on the extremities. The patient subsequently developed left inguinal lymph node metastasis, and finally presented a single brain metastasis at the left occipital, BRAF mutated 3.5 years after the primary diagnosis. The patient was deceased 14 months after brain metastasis diagnosis.

**Specimen ID:** MBM-40

Male patient with a diagnosis of stage IA nodular melanoma on the trunk at 54 years old. The patient presented a single brain metastasis at the left parietal, NRAS mutated 11 months after the initial diagnosis. The patient was deceased 11 months after brain metastasis diagnosis.

**Specimen ID:** MBM-41

Female patient with occult melanoma at 62 years old due to brain metastasis at right frontal-parietal, NRAS mutated. The patient was deceased 4 months after brain metastasis diagnosis.

**Specimen ID:** MBM-42

Male patient with stage IIIC melanoma on the foot at 17 years old. The patient then developed in transit right lower leg metastasis followed by bone metastasis. Finally presented two brain metastases at right cerebellar and right frontal lobe, BRAF mutated 14 years after initial diagnosis. The patient died 10 months after brain metastasis diagnosis.

**Specimen ID:** MBM-43

43-year-old male diagnosed with stage IB superficial spreading melanoma on the trunk who subsequently presented lung metastasis. The patient finally presented a single brain metastasis at the frontal lobe, NRAS mutated 4.3 years after initial diagnosis. The patient was deceased 10 months after brain metastasis diagnosis.

**Specimen ID:** MBM-44

Male patient with a diagnosis of stage IA superficial spreading melanoma on the trunk at 68 years old. The patient subsequently developed regional lymph node metastasis followed by spleen metastasis. The patient finally developed a single brain metastasis at the left occipital lobe, BRAF mutated, 4 years after the initial diagnosis. The patient was deceased 12 months after brain metastasis diagnosis.

**Specimen ID:** MBM-45

40-year-old female patient diagnosed with stage IB lentigo maligna melanoma. The patient then presented multiple simultaneous brain, right chest, small intestine, and multiple lymphadenopathies metastases. The multiple brain metastases were located in the right frontal, and cerebellum, BRAF mutated.

**Specimen ID:** MBM-46

75-year-old male patient diagnosed with stage IA superficial spreading melanoma on mid-chest. The patient subsequently presented lumbar spine and retroperitoneal metastasis. The patient then developed four brain metastases at the right frontal lobe and right temporoparietal, BRAF mutated 4.4 years after the initial diagnosis. The patient was deceased 3 months after brain metastases diagnosis.

**Specimen ID:** MBM-47

Male patient diagnosed with melanoma at 56 years old with a simultaneous brain, liver, and lung metastasis at initial diagnosis. The patient presented multiple brain metastases at right frontal-parietal, right occipital, right cerebellar, and left parietal, BRAF mutated. The patient died 11 months after brain metastasis diagnosis.

**Specimen ID:** MBM-48

Female patient diagnosed with melanoma at 50 years old. The patient subsequently developed shoulder, abdomen, chest, and back metastasis. 6 years after initial diagnosis the patient developed a single brain metastasis at the left parietal, NRAS mutated. The patient was deceased 3 years after brain metastasis diagnosis.

**Specimen ID:** MBM-49

49-year-old male patient diagnosed with stage IB superficial spreading melanoma on extremities. The patient subsequently developed left knee, left groin lymph node followed by lung metastasis. Finally presenting two brain metastases at the left hippocampus and left frontal lobe, NRAS mutated 3 years after initial diagnosis. The patient died 2 years after brain metastasis diagnosis.

**Specimen ID:** MBM-50

Male patient diagnosed with melanoma on the trunk at age 41. The patient developed cutaneous, lung, liver, and abdomen metastasis with lymph node metastasis. The patient then developed a single brain metastasis at right temporal, BRAF mutated 3 years after initial diagnosis. The patient died 16 months after brain metastasis diagnosis.

**Specimen ID:** MBM-51

Male patient diagnosed with occult melanoma at 76 years old. The patient developed pulmonary hillium followed by liver metastasis. 15 months after the initial diagnosis the patient presented a single brain metastasis at the left frontal lobe. The patient died 1 month after brain metastasis diagnosis.

**Specimen ID:** MBM-52

Male patient diagnosed with occult melanoma due to lung metastasis, at 71 years old. He then presented a single brain metastasis at right occipital, d-WT 19 months after initial diagnosis. The patient died 11 months after brain metastasis diagnosis.

**Specimen ID:** MBM-53

51-year-old male patient diagnosed with stage IIB melanoma with simultaneous brain and lung metastasis followed by duodenal adenocarcinoma metastasis at initial diagnosis. He then presented a single brain metastasis at right parietal, NRAS mutated 3 years after the initial diagnosis. The patient died 7 years after brain metastasis diagnosis.

**Brain metastasis (BM) specimens with unknown or uncertain diagnosis**

**Specimen ID:** BM-01

Female patient, with a history of breast and lung cancer at 54 years old. The patient developed brain metastases at 58 years old. Anatomic-pathology evaluation compatible with lung cancer brain metastasis. No confirmatory immunohistochemistry evaluation was performed.

**Specimen ID:** BM-02

Female patient, with a history of invasive breast cancer, but no history of lung cancer. The patient developed brain metastases at 68 years old. Immunohistochemistry evaluation compatible with lung cancer brain metastasis (TTF-1 positive, Napsin-A positive).

**Specimen ID:** BM-03

Female patient with a history of non-small cell lung cancer at 43 years, invasive breast cancer and, non-Hodgkin's lymphoma at 71 years old. At 80 years old, she presented a brain metastasis in the frontal lobe, compatible with metastatic pulmonary adenocarcinoma.

**Specimen ID:** BM-04

64 years old female patient with a history of pulmonary adenocarcinoma. The patient developed brain metastases histologically compatible with metastatic pulmonary adenocarcinoma, but lacking confirmatory immunohistochemistry, CK7positive/CK20 negative, TTF-1 negative, Napsin-A negative.
