# Supplementary material for: Epigenetic profiling for the molecular classification of metastatic brain tumors
Source: Nat Commun. 2018 Nov 6;9:4627. doi: 10.1038/s41467-018-06715-y (PMC6219520; doi:10.1038/s41467-018-06715-y)
Supplement: Supplementary file 3 — Description of Additional Supplementary Files [file 41467_2018_6715_MOESM3_ESM.pdf]

Description of Additional Supplementary Files

File Name: Supplementary Data 1
Description: Clinical and demographic data of 94 patients with a diagnosis of brain metastasis from lung cancer, breast cancer, and cutaneous melanoma.

File Name: Supplementary Data 2
Description: Cumulative variance of DNA methylation differences between primary brain tumors and brain metastases in a principal component analysis (PCA) using ten randomly selected HM450K probe sets.

File Name: Supplementary Data 3
Description: Genomic regions evaluated by targeted quantitative methylation-specific PCR (qMSP) to generate the brain metastasis DNA methylation classifier A (BrainMETH class A; n=12). Each region includes information about the primer sequences with melting temperatures for qMSP, Illumina HM450K probe identifier, genomic region coordinates for the hg19 genome assembly, distance to the nearest gene, and qMSP performance. Additionally, primer sequences for locus-specific bisulfite sequencing are provided for genomic regions with poor qMSP performance.

File Name: Supplementary Data 4
Description: Significantly differentially methylated genomic regions among the three types of brain metastases assessed by one-way ANOVA with a threshold of adjusted Bonferroni corrected P-value =0.05 (n=31,818).

File Name: Supplementary Data 5
Description: Gene ontology and pathway enrichment analyses of specific hypomethylated regions of the three types of brain metastases using the Genomic Regions Enrichment of Annotations Tool (GREAT).

File Name: Supplementary Data 6
Description: Genomic regions evaluated by qMSP to generate the BrainMETH class B (n=9). Each region includes information about the primer sequences with melting temperatures for qMSP, Illumina HM450K probe identifier, genomic region coordinates for the hg19 genome assembly, distance to the nearest gene, and qMSP performance. Additionally, primer sequences for locus-specific bisulfite sequencing are provided for genomic regions with poor qMSP performance.

File Name: Supplementary Data 7
Description: Significantly differentially methylated genomic regions among the subtypes of breast cancer brain metastases assessed by one-way ANOVA with a threshold of false discovery rate corrected P-value =0.0005 (n=409).

File Name: Supplementary Data 8
Description: Clustering information for each differentially methylated genomic regions among the subtypes of breast cancer brain metastases (n=409). DNA methylation level (β-value) is provided for each specimen included in the analysis (n=24).

File Name: Supplementary Data 9

Description: Genomic regions specifically associated with the subtypes of breast cancer brain metastases identified using the nearest shrunken centroid algorithm (n=126).

File Name: Supplementary Data 10

Description: Genomic regions evaluated by qMSP to generate the BrainMETH class C (n=10). Each region includes information about the primer sequences with melting temperatures for qMSP, Illumina HM450K probe identifier, genomic region coordinates for the hg19 genome assembly, distance to the nearest gene, and qMSP performance. Additionally, primer sequences for locus-specific bisulfite sequencing are provided for genomic regions with poor qMSP performance.
